# Benefits of 6-weeks high-intensity circuit training with or without blood flow restriction on physical fitness in active males: A randomized controlled trial

**DOI:** 10.1016/j.jesf.2026.200525

**Published:** 2026-09-20

**Authors:** Yun-Rong Yang, Chen-Meng Goh, Chin-Yu Lin, Chu Chen, Chi-Hsueh Pan, Ching-Feng Cheng

**Affiliations:** a Department of Physical Education and Sports Sciences, National Taiwan Normal University, No. 162, Section 1, Hoping E. Rd, Da-an District, Taipei, 106, Taiwan; b Sports Performance Lab, Department of Sport and Kinesiology, National Taiwan Normal University, No. 88, Section 4, Tingzhou Rd., Wen-shan District, Taipei, 116, Taiwan; c Department of Sport and Kinesiology, National Taiwan Normal University, No. 88, Section. 4, Tingzhou Rd., Wen-shan District, Taipei, 116, Taiwan

**Keywords:** High-intensity interval training, Local hypoxia, Peripheral fatigue, Physical activity, Weight-bearing exercise

## Abstract

**Objective:**

To investigate the effects of a 6-weeks 7-min workout (7 MW), with and without blood flow restriction (BFR), on physical fitness.

**Methods:**

Thirty-three active healthy males (age: 23.3 ± 2.6 yr; body mass index: 22.5 ± 1.9 kg m^−2^) were randomly assigned to 7 MW, 7 MW-BFR (150 mmHg cuff pressure applied to proximal thighs), or control (CON) groups. 7 MW, comprising 12 bodyweight exercises, was performed 3 times per week, with one set during weeks 1–3 and two sets during weeks 4–6. Outcomes included body fat percentage (%BF), thigh circumference (TC), maximal oxygen uptake (VO_2_max), isometric mid-thigh pull variables (peak [PF] and mean [MF] force, and maximal rate of force development [RFDmax]), and countermovement jump (CMJ) height.

**Results:**

Adjusting for baseline values, post-intervention VO_2_max was higher in 7 MW (*p* < 0.001, 95% CI: 2.1–5.7 mL kg^−1^·min^−1^) and 7 MW-BFR (*p* = 0.002, 95% CI: 1.3–4.9 mL kg^−1^·min^−1^) than in CON. PF and MF were greater in both training groups than in CON, with larger effects in 7 MW-BFR (PF: *p* < 0.001, *d* = 1.21, 95% CI: 155–451 N; MF: *p* < 0.001, *d* = 1.15, 95% CI: 142–448 N) than in 7 MW (PF: *p* = 0.030, *d* = 0.63, 95% CI: 17–315 N; MF: *p* = 0.026, *d* = 0.68, 95% CI: 23–332 N). No significant changes were observed in %BF, TC, RFDmax, or CMJ height.

**Conclusions:**

The 7 MW-based program can improve aerobic capacity and muscular strength in active males, and BFR may further augment strength gains.

## 1 Introduction

High-intensity interval training (HIIT) is an effective and efficient method to improve aerobic and muscular fitness, and overall health.^1^, ^2^, ^3^ Beyond conventional running- or cycling-based HIIT, the recently popular high-intensity circuit training (HICT), which utilizes bodyweight exercises within a HIIT framework, may offer a cost-efficient, minimal-equipment, and easily accessible (e.g., in-place) approach to enhancing overall health.^4^ Interestingly, although HICT is conveniently implemented in various settings, studies exploring its long-term effects on physical fitness, especially in active adults, remain limited and show inconsistent findings. Several studies have reported that HICT could improve aerobic capacity^5^ and muscular fitness,^6^^,^^7^ and reduce body fat (BF)^5^^,^^8^ in active adults, whereas other studies have shown conflicting results.^9^^,^^10^ These discrepancies may be partially explained by the inadequate training stimulus offered by bodyweight exercises for active adults,^11^ as well as differences in training protocols, such as the training volume, exercise selection, and the design of control group. Therefore, further study is warranted to clarify the effects of HICT on physical fitness and to identify the most effective training regimens, especially within active populations.

The American College of Sports Medicine (ACSM)^12^^,^^13^ recommends adults engage in moderate-intensity (64–76% maximal heart rate [HRmax], ≥150 min/week) or vigorous-intensity (77–95% HRmax, ≥75 min/week) cardiorespiratory exercise, along with resistance exercises (≥2 sessions/week) to improve physical fitness. However, perceived lack of time, space, and equipment is a common barrier to exercise participation.^14^^,^^15^ The 7-min workout (7 MW)-based HICT program requiring minimal equipment, has been proposed as a time-efficient alternative.^4^ Despite its practicality, a single bout of 7 MW elicits only 76.6% (mean) and 86.6% (peak) HRmax in active adults,^11^ corresponding to the lower range of vigorous intensity, which may limit cardiovascular adaptations.^12^^,^^16^ Moreover, the reliance on bodyweight in the 7 MW may fail to provide sufficient resistance (i.e., 80% of one repetition maximum [1RM]) necessary to optimize muscular strength.^13^ To address this limitation, strategies aimed at augmenting the training stimulus, such as the incorporation of external loading, may be considered for active adults performing the 7 MW.^12^^,^^17^

Blood flow restriction (BFR) training utilizes an inflated cuff to partially occlude blood flow to working muscle groups, thereby increasing the internal workload when the external workload remains consistent.^18^^,^^19^ While the mechanisms underlying skeletal muscle growth remain incompletely elucidated, preview studies indicated that factors such as mechanical tension and metabolic stress may play significant roles in facilitating adaptations related to muscle strength and hypertrophy.^20^^,^^21^ The venous occlusion caused by BFR during aerobic or resistance exercise may accelerate peripheral fatigue and increase type II fiber recruitment, thereby facilitating increased muscle activation that contributes to strength gains and hypertrophic adaptations.^18^^,^^19^^,^^22^^,^^23^ Moreover, metabolite accumulation (e.g., lactate [La] or H^+^) induced by BFR may indirectly increase local muscular fatigue and motor unit recruitment.^18^^,^^19^ Additionally, a rise in HR caused by venous occlusion, which reduces stroke volume, could increase cardiac structure adaptation.^24^^,^^25^ Furthermore, partial arterial occlusion creates a local hypoxic environment, which could activate hypoxia-inducible factor-1α, upregulating angiogenic mRNAs expression,^24^^,^^26^ and vascular shear stress attributed to deflation could improve angiogenesis.^19^ Previous studies reported that maximal effort-based training (i.e., sprint interval training [SIT] and repeated-sprint training [RST]) could further improve VO_2_max in healthy males^27^ and lower body strength in basketball player^28^ when incorporating BFR. Consequently, BFR might be an external means of increasing cardiovascular and muscular demands during 7 MW, thereby enhancing training adaptations.

Therefore, this study aimed to examine the effects of the 7 MW with or without BFR on physical fitness in active adults. We hypothesized that the 7 MW can improve aerobic capacity, while the 7 MW combined with BFR can elicit further aerobic and muscular adaptations in active adults due to the greater physiological stimulus induced by BFR.

## 2 Methods

### 2.1 Experimental design

This study employed a randomized controlled trial design. During the first visit, arterial occlusion pressure (AOP) was assessed utilizing the Kaatsu Master 2.0 training cuff (Sato Sports Plaza Ltd., Japan) in conjunction with a hand-held Doppler probe (MD6, Hokanson, USA).^29^ After familiarization, participants underwent the physical fitness measurements, including body composition, muscular fitness tests (i.e., the countermovement jump [CMJ] test and the isometric mid-thigh pull [IMTP] test), and a graded exercise test (GXT). Subsequently, participants were randomly allocated to 7 MW, 7 MW with BFR (7 MW-BFR), or a control (CON) group using a random draw. The 7 MW and the 7 MW-BFR performed three sessions weekly for 6 weeks, while the CON did not undergo any intervention. After intervention, the identical physical fitness measurements were administered as the post-intervention test (Fig. 1).

**Fig. 1:**
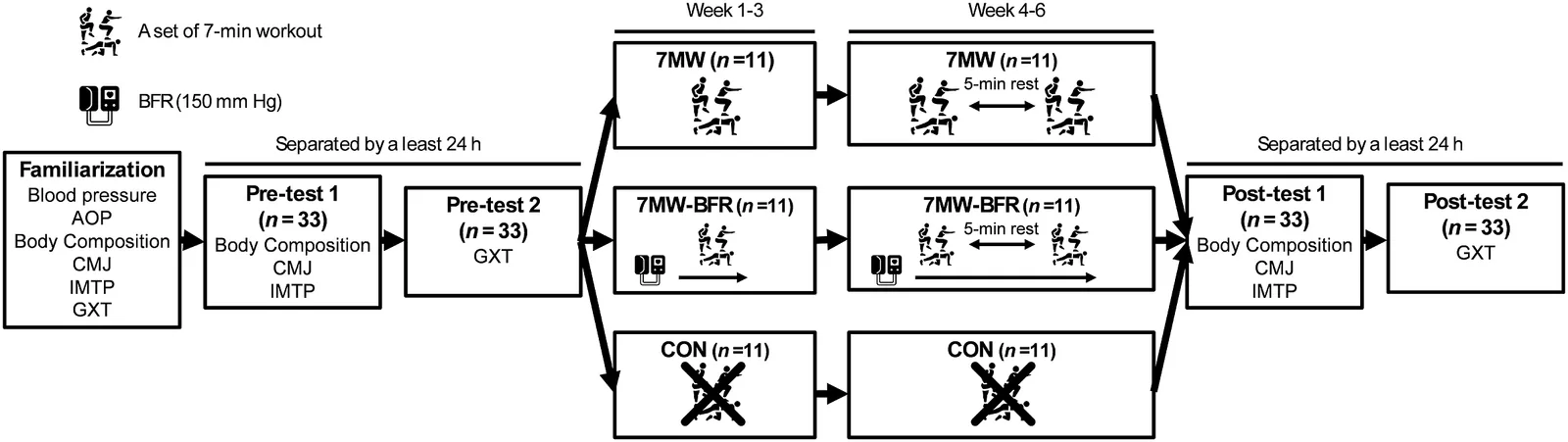
Schematic representation of the study protocol. 7 MW: 7-min workout group; BFR: blood flow restriction; 7 MW-BFR: 7 MW with BFR group; CON: control group; AOP: arterial occlusion pressure measurement; CMJ: countermovement jump; IMTP: isometric mid-thigh pull test; GXT: graded exercise test.

### 2.2 Participants

Based on a previous meta-analysis that identified a moderate effect size of BFR training on aerobic capacity,^30^ an *a priori* analysis was conducted using G*Power software (*α* = 0.05, *β* = 0.20, effect size = 0.35), indicating that a sample size of *n* = 8 in each group was required. Thirty-three recreationally active males (Tier 1)^31^ participated in this study after providing written informed consent (Table 1). All participants engaged in recreational activities (running, resistance training, basketball, tennis, badminton, and volleyball) at least twice per week before participation in the present study, and had no medical history of musculoskeletal injuries, cardiovascular diseases or blood pressure >130/80 mmHg (systolic/diastolic). Participants were instructed to maintain their habitual physical activity throughout the intervention period and to avoid engaging in any additional vigorous exercise or structured training programs. The intervention took place during the academic semester. All experimental procedures were conducted in the Sports Performance Lab at National Taiwan Normal University, Taipei, Taiwan. This study was approved by the local Research Ethics Committee of National Taiwan Normal University (REC no. 202112HM002).

**Table 1:** Participant characteristics in the 7-min workout (7 MW), 7 MW with blood flow restriction (7 MW-BFR), and control (CON) groups at baseline.

	7 MW (*n* = 11)	7 MW-BFR (*n* = 11)	CON (*n* = 11)
Age (yr)	23.1 (2.7)	23.1 (3.3)	23.7 (1.9)
Height (m)	1.74 (0.06)	1.77 (0.04)	1.76 (0.05)
Body mass (kg)	66.8 (8.7)	70.2 (9.1)	72.0 (3.9)
SBP (mm Hg)	119 (9)	119 (7)	121 (7)
DBP (mm Hg)	66 (9)	61 (7)	64 (6)
AOP (mm Hg)	490 (33)	466 (66)	456 (74)

Data are presented as mean (standard deviation [SD]). SBP: systolic blood pressure; DBP: diastolic blood pressure; AOP: arterial occlusion pressure.

### 2.3 Arterial occlusion pressure measurement

The AOP testing procedures followed those described in a previous study.^29^ Before the AOP test, participants were asked to lie supine for 10 min. Then, a Kaatsu training cuff (5 cm wide) was applied to the most proximal portion of the right thigh. A hand-held Doppler probe placed over the right tibialis posterior artery was used to measure the AOP. Initially, the cuff was inflated to 40 mm Hg for 30 s, followed by a 10-s deflation. The cuff pressure was then increased to participants’ systolic blood pressure level and maintained for 30 s, followed by another 10-s deflation. Thereafter, the cuff pressure was increased by 40 mm Hg (30-s inflation followed by 10-s deflation) until arterial blood flow was no longer detectable. Subsequently, the cuff pressure was decreased by 10 mm Hg increments every 10 s until arterial blood flow was detected again. The lowest cuff pressure at which the pulse was not detected was determined as the AOP. However, due to equipment limitations, the maximum cuff pressure achievable was 500 mm Hg. Therefore, when the cuff pressure reached 500 mm Hg, and participants still had a detectable pulse, their AOP was recorded as 500 mm Hg. This phenomenon was observed in 10 participants within the 7 MW group and in 7 participants within the 7 MW-BFR group.

### 2.4 Physical fitness assessments

After assessment of body mass with a digital scale (Tanita HD-319, Tanita Corporation, Japan), an experienced researcher then assessed their three-site skinfold thickness (chest, abdomen, and thigh) using Lange skinfold calipers (Beta Technology Inc., CA, USA) and thigh circumference (TC). Each skinfold site was measured twice separated by 1-min. The mean of these two measurements was then utilized to calculate BF percentage (%BF) according to the three-site formula outlined in the ACSM guidelines.^32^ After the skinfold thickness measurements, participant was asked to stand on a step with one leg and to extend the measured leg in a relaxed position in order to evaluate the TC. The TC at the midpoint between the inguinal crease and the proximal border of the patella was measured using a body tape measure.^32^ Both legs were measured twice, separated by 1 min, and the average value was used for analysis. Body mass index (BMI) was calculated as body mass (kg) divided by height squared (m^2^).

Thereafter, the participants performed a 5-min standardized warm-up (100 W; 60 rpm) on a Cyclus 2 ergometer (RBM Elektronik-Automation, Germany). Then, the participants performed three CMJs without arm swing on a force plate (Ballistic Measurement System, Innervations Inc., Australia) separated by 10 s of rest.^33^ The highest attempt was used for analysis. After the CMJ test and a 2-min rest, the participants performed three IMTP trials with 2-min rest intervals. The IMTP test was initiated from a deadlift position, with the knees flexed at 125° and the hips flexed at 145°.^33^ Participants were instructed to pull a fixed bar upward with maximal effort for 5 s. The highest peak force (PF) recorded was used for data analysis. Additionally, the 5-s mean force (MF) and the maximal rate of force development (RFDmax), defined as the highest 30-ms slope on the force-time curve, were calculated.

During the second visit, participants performed the cycling GXT after the standardized warm-up. Respiratory gas data were collected using the Metamax 3B metabolic measurement system (Cortex, Germany), while HR was monitored using a Polar RS800 wireless HR monitor (Polar Electro Inc., Finland). The GXT protocol followed the guidelines from previous research,^34^ started at 25 W with an increment of 15 W every 3 min. Once the respiratory exchange ratio (RER) reached 1.0, the workload increment was increased to 30 W min^−1^ until volitional exhaustion. According to previous studies,^35^^,^^36^ exhaustion during the GXT was defined as meeting at least four of the following six criteria: (1) despite strong verbal encouragement, the pedaling cadence dropped by more than 10 rpm below the self-selected rate (60, 70, 80, 90 rpm) for over 10 s; (2) RER exceeded 1.2; (3) HR surpassed the predicted maximum HR (220 − age); (4) oxygen uptake plateaued despite increasing workload (<150 ml min^−1^ increase); (5) the rating of perceived exertion exceeded 17 (Borg 6–20 scale); and (6) participants reported subjective fatigue, exhaustion, or an inability to continue the test. Both absolute and relative VO_2_max were determined as the highest 30-s average value during the test. Capillary blood [La] was measured before and 5 min post-GXT via right earlobe puncture using a Lactate Pro2 analyzer (Arkray, Inc., Japan).

### 2.5 Training intervention

Participants in 7 MW and 7 MW-BFR groups underwent three weekly training sessions for 6 weeks, separated by at least 48 h. Each session included a standardized warm-up and the 7 MW with or without BFR. Table 2 shows a set of 7 MW comprising 3 static exercises and 9 dynamic exercises. Participants performed each dynamic exercise as many times as possible at maximal movement velocity, with each exercise lasting 30 s and a 10-s rest between exercises.^4^^,^^6^^,^^8^^,^^9^ Over 6 weeks, participants performed 1 set of 7 MW initially, progressing to 2 sets with 5-min rest in weeks 4–6. For the 7 MW-BFR group, vascular occlusion was applied to both thighs using Kaatsu cuffs inflated to 150 mmHg (31.8 ± 4.6%AOP), which was identified as the maximum tolerable pressure in our pilot study. This occlusion was maintained continuously throughout the 7 MW and during the 5-min inter-set rests, with total occlusion durations of 470 s and 1240 s in the initial and final three weeks, respectively. The average number of repetitions for each dynamic exercise completed during each set, and the average session-rating of perceived exertion (s-RPE; Borg 0–10 scale) for the first and second 3 weeks were calculated for subsequent analysis.^37^

**Table 2:** The 7-min workout (7 MW) protocol.

Exercise order	Exercise name	Exercise type
1	Jumping jacks	Dynamic
2	Wall sit	Static
3	Push-ups	Dynamic
4	Crunch	Dynamic
5	Step-ups	Dynamic
6	Squats	Dynamic
7	Triceps dips	Dynamic
8	Plank	Static
9	High knees running	Dynamic
10	Lunges	Dynamic
11	Push-ups and rotation	Dynamic
12	Side plank (15 s for both sides)	Static

### 2.6 Statistical analysis

All data are expressed as mean ± standard deviation, unless indicated otherwise. All analyses were conducted utilizing SPSS 29.0 software (IBM Corp., USA). Normality of the sample distribution was confirmed with the Shapiro–Wilk test. Post-intervention physical fitness variables were analyzed across groups utilizing analysis of covariance (ANCOVA), incorporating the respective pre-intervention measurements as covariates. Partial eta squared (ηp^2^) was reported to evaluate the effect size for the main effects of ANCOVA models. The ηp^2^ values of 0.01, 0.06, and 0.14 indicated small, moderate, and large effects, respectively.^38^ The average s-RPE and number of dynamic exercises completed were compared using 2 × 2 (group × time) and 2 × 3 (group × set) two-way mixed analysis of variance (ANOVA), respectively. A least significant difference (LSD) *post hoc* test was employed when a significant interaction effect was detected, as recommended for testing individual hypotheses.^39^ Cohen's *d* was calculated as the effect size, and *d* values of 0.2, 0.5, and 0.8 indicated small, moderate, and large effects, respectively,^38^ and the 95% confidence intervals (CI) of the mean differences derived from paired comparisons were also computed. The statistical significance level was set at *α* < 0.05.

## 3 Results

### 3.1 Physical fitness

A total of 33 participants were randomly allocated to three groups (*n* = 11 per group). All participants completed the intervention and were included in the final analysis (Fig. 2). The raw physical fitness data at pre- and post-intervention are presented in Table 3. After adjustment for pre-intervention values, a significant main effect of group was observed for both post-intervention absolute and relative VO_2_max (absolute: *F* = 6.574, *p* = 0.004, ηp^2^ = 0.20; relative: *F* = 10.815, *p* < 0.001, ηp^2^ = 0.43), whereas no significant group effect was found for post-intervention [La] following the GXT (*F* = 1.314, *p* = 0.284, ηp^2^ = 0.08). Both adjusted post-intervention absolute and relative VO_2_max were significantly greater in 7 MW (absolute: *p* = 0.013, *d* = 0.65, 95% CI: 0.05–0.40 L min^−1^; relative: *p* < 0.001, *d* = 0.78, 95% CI: 2.1–5.7 mL kg^−1^·min^−1^) and 7 MW-BFR (absolute: *p* = 0.001, *d* = 0.84, 95% CI: 0.12–0.45 L min^−1^; relative: *p* = 0.002, *d* = 0.65, 95% CI: 1.3–4.9 mL kg^−1^·min^−1^) compared with CON (Fig. 3A and B).

**Fig. 2:**
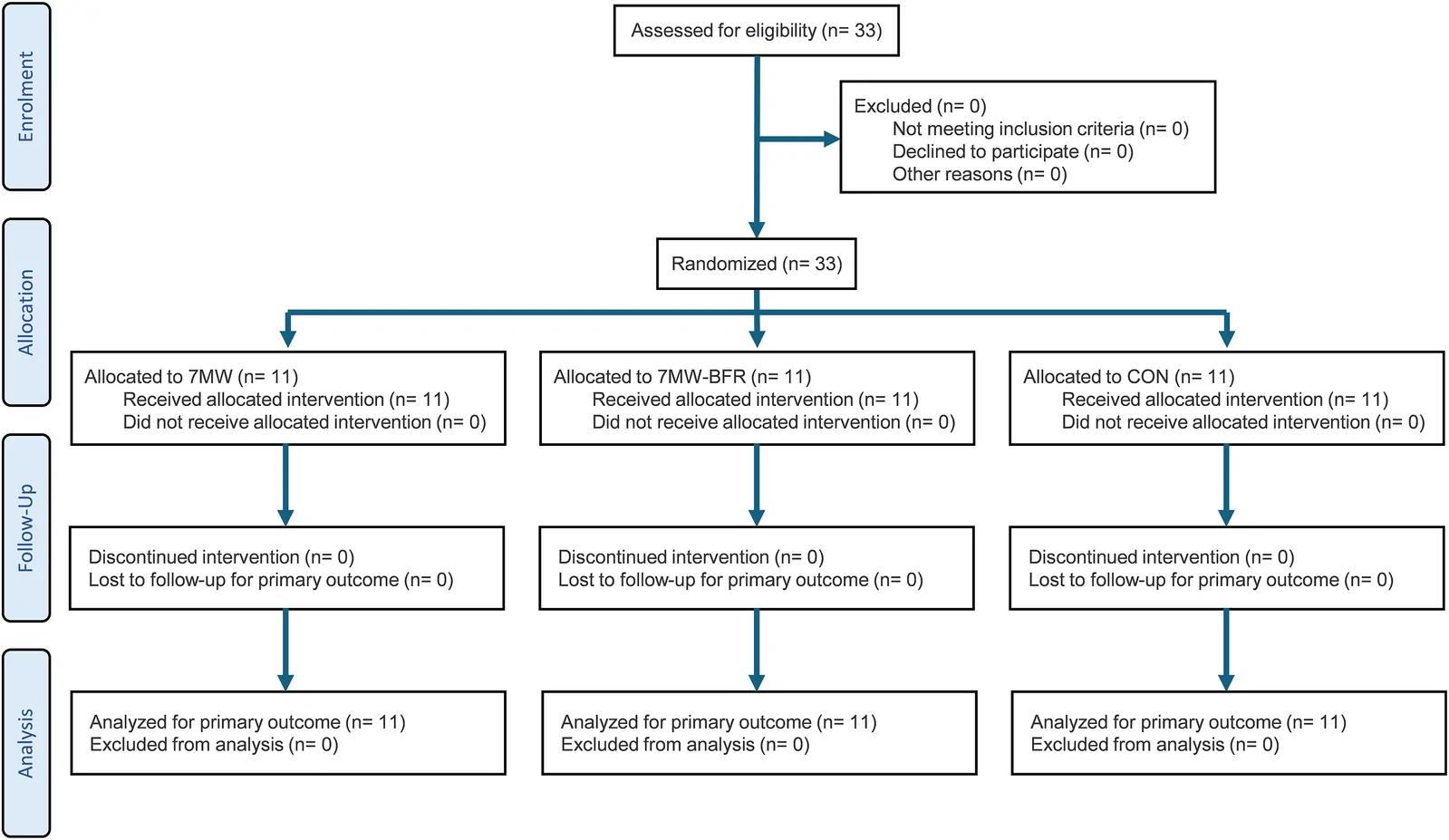
CONSORT Flow Chart of the study. 7 MW: 7-min workout group; BFR: blood flow restriction; 7 MW-BFR: 7 MW with BFR group; CON: control group.

**Table 3:** The comparison of graded exercise test, muscular strength and power test, and body composition measurement parameters before and after a 6-week intervention in the 7-min workout (7 MW), 7 MW with blood flow restriction (7 MW-BFR), and control (CON) groups.

	7 MW (*n* = 11)		7 MW-BFR (*n* = 11)		CON (*n* = 11)	
	Pre-test	Post-test	Pre-test	Post-test	Pre-test	Post-test
Cardiovascular fitness						
Absolute VO_2_max	3.34 (0.27)	3.52 (0.31)	3.42 (0.30)	3.66 (0.33)	3.75 (0.41)	3.67 (0.39)
Relative VO_2_max	50.5 (3.1)	53.6 (5.8)	49.3 (5.5)	51.7 (5.4)	52.2 (5.2)	51.2 (4.6)
PRE-[La]	1.2 (0.3)	1.3 (0.4)	1.2 (0.3)	1.2 (0.2)	1.3 (0.2)	1.1 (0.3)
POST-[La]	7.4 (2.1)	8.3 (2.3)	8.7 (2.5)	8.6 (2.8)	9.1 (2.5)	8.2 (2.3)
Muscular fitness						
CMJ	40.1 (6.1)	40.0 (6.0)	38.2 (5.9)	39.8 (5.1)	39.8 (5.9)	38.7 (7.6)
PF	2006 (386)	2078 (320)	2040 (417)	2236 (293)	2147 (304)	1999 (227)
MF	1808 (362)	1885 (303)	1860 (384)	2035 (293)	1952 (340)	1797 (244)
RFDmax	7581 (2646)	6993 (2321)	7133 (2775)	8477 (2712)	9189 (3821)	8285 (4680)
Body composition						
BM	66.8 (8.7)	66.4 (9.0)	70.2 (9.1)	71.5 (9.4)	72.0 (3.9)	71.8 (4.1)
BMI	22.0 (1.6)	21.8 (1.7)	22.3 (2.3)	22.7 (2.3)	23.2 (1.5)	23.1 (1.5)
TC	54.1 (3.7)	53.4 (3.4)	54.8 (4.7)	54.9 (4.6)	55.6 (2.6)	55.5 (2.9)
Chest ST	9.3 (4.9)	8.7 (4.5)	9.7 (3.3)	8.0 (2.8)	9.4 (4.7)	10.7 (6.1)
Abdominal ST	15.4 (6.6)	15.3 (4.5)	16.3 (5.6)	16.0 (6.5)	16.5 (6.0)	20.0 (8.8)
Thigh ST	12.1 (4.5)	11.6 (3.9)	14.5 (4.3)	13.7 (5.3)	12.8 (4.8)	13.1 (3.7)
Sum of ST	36.8 (15.3)	35.4 (14.8)	40.5 (12.3)	37.6 (13.8)	38.8 (13.7)	43.8 (17.7)
%BF	10.3 (4.6)	9.9 (4.6)	11.4 (3.7)	10.6 (4.0)	10.9 (4.0)	12.3 (5.1)

Data are presented as mean (standard deviation [SD]). VO_2_max: maximal oxygen uptake (absolute: L·min^−1^; relative: mL·kg^−1^·min^−1^); PRE-[La]: lactate concentration before graded exercise test (GXT) (mmol·L^−1^); POST-[La]: lactate concentration after GXT (mmol·L^−1^); CMJ: countermovement jump height (cm); PF: peak force from isometric mid-thigh pull (IMTP) (N); MF: mean force from IMTP (N); RFDmax: maximal rate of force development from IMTP (N·s^−1^); BM: body mass (kg); BMI: body mass index (kg·m^−2^); TC: thigh circumferences (cm); ST: skinfold thickness (mm); %BF: body fat percentage (%).

**Fig. 3:**
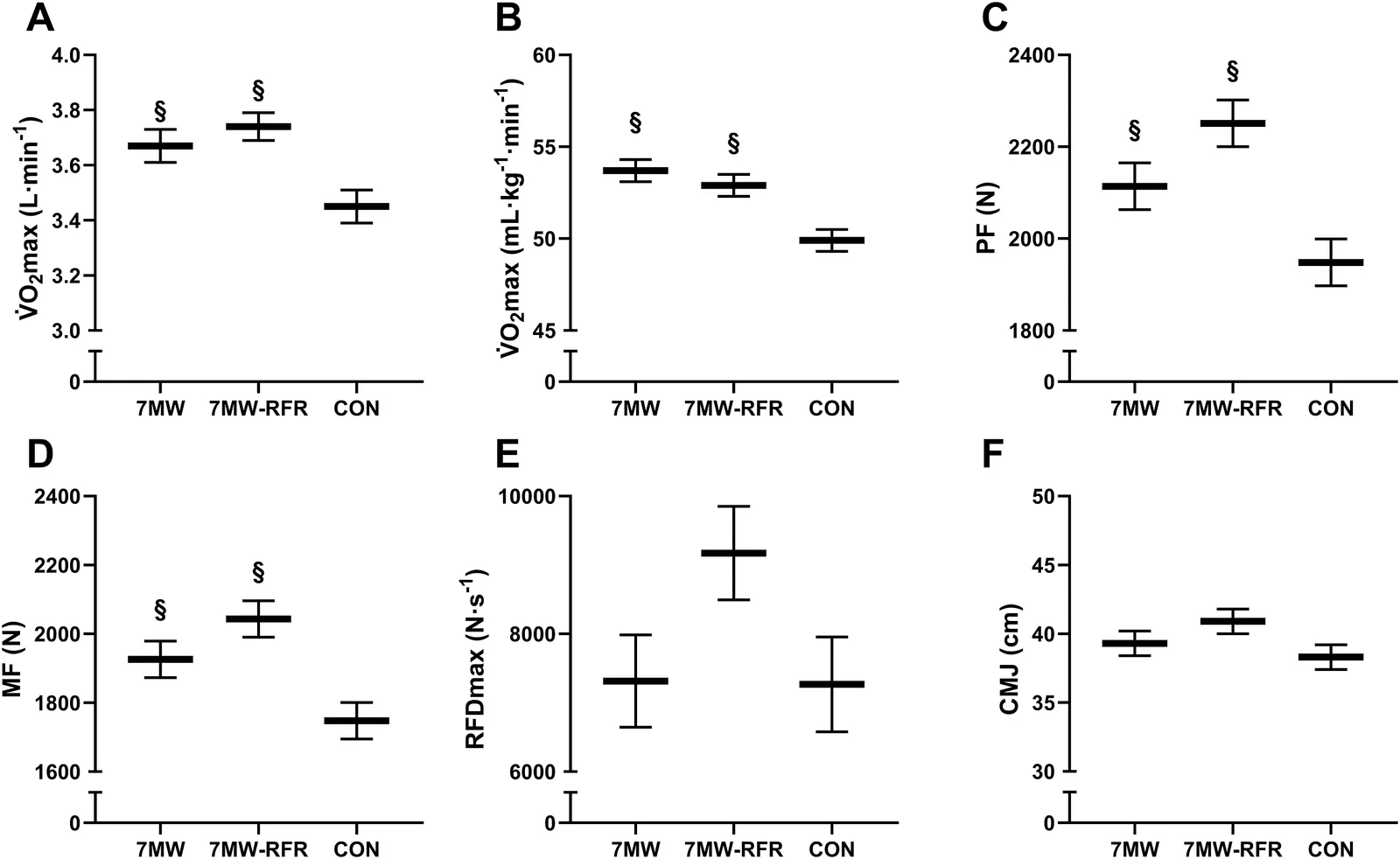
Comparison of the adjusted values of absolute (A) and relative (B) VO_2_max and muscular fitness (C–F) after a 6-week intervention in the 7-min workout (7 MW), 7 MW with blood flow restriction (7 MW-BFR), and control (CON) groups. Data are presented as adjusted mean ± standard error (SE). VO_2_max: maximal oxygen uptake; CMJ: countermovement jump; PF: peak force from isometric mid-thigh pull tests (IMTP); MF: mean force from IMTP; RFDmax: maximal rate of force development from IMTP; %BF: body fat percentage. ^§^*P* < 0.05, compared with CON.

After adjustment for pre-intervention values, a significant main effect of group was observed for post-intervention PF (*F* = 8.745, *p* = 0.001, ηp^2^ = 0.38; Fig. 3C) and MF (*F* = 7.862, *p* = 0.002, ηp^2^ = 0.35; Fig. 3D), whereas no significant group effect was found for post-intervention RFDmax (*F* = 2.534, *p* = 0.097, ηp^2^ = 0.15; Fig. 3E) and CMJ height (*F* = 1.948, *p* = 0.161, ηp^2^ = 0.12; Fig. 3F). The adjusted post-intervention PF was significantly greater in 7 MW (*p* = 0.030, *d* = 0.63, 95% CI: 17–315 N) and 7 MW-BFR (*p* < 0.001, *d* = 1.21, 95% CI: 155–451 N) compared with CON. Similarly, the adjusted post-intervention MF was also significantly greater in 7 MW (*p* = 0.026, *d* = 0.68, 95% CI: 23–332 N) and 7 MW-BFR (*p* < 0.001, *d* = 1.15, 95% CI: 142–448 N) compared with CON. Notably, although no significant differences were observed between the 7 MW and 7 MW-BFR groups for PF (*p* = 0.068) and MF (*p* = 0.125), the 7 MW-BFR group demonstrated greater percentage improvements in PF (+12.0 ± 15.6% vs +4.5 ± 8.5%) and MF (+11.3 ± 13.0% vs +5.3 ± 10.3%) than the 7 MW group, accompanied by larger effect sizes relative to CON.

The adjusted body composition values at post-intervention are presented in Fig. 4. After adjustment for pre-intervention values, no significant main effect of group was found for post-intervention TC (*F* = 1.614, *p* = 0.216, ηp^2^ = 0.10), chest skinfold thickness (*F* = 2.767, *p* = 0.079, ηp^2^ = 0.16), thigh skinfold thickness (*F* = 0.469, *p* = 0.630, ηp^2^ = 0.03), sum of skinfold thickness (*F* = 3.142, *p* = 0.058, ηp^2^ = 0.18), %BF (*F* = 3.163, *p* = 0.057, ηp^2^ = 0.18), body mass (*F* = 2.991, *p* = 0.066, ηp^2^ = 0.17), and BMI (*F* = 3.138, *p* = 0.058, ηp^2^ = 0.18), except for abdominal skinfold thickness (*F* = 4.119, *p* = 0.027, ηp^2^ = 0.22). The adjusted post-intervention abdominal skinfold thickness was significantly lower in 7 MW (*p* = 0.026, *d* = 0.47, 95% CI: −6.7 to −0.5 mm) and 7 MW-BFR (*p* = 0.015, *d* = 0.53, 95% CI: −7.0 to −0.8 mm) compared with CON (Fig. 4E).

**Fig. 4:**
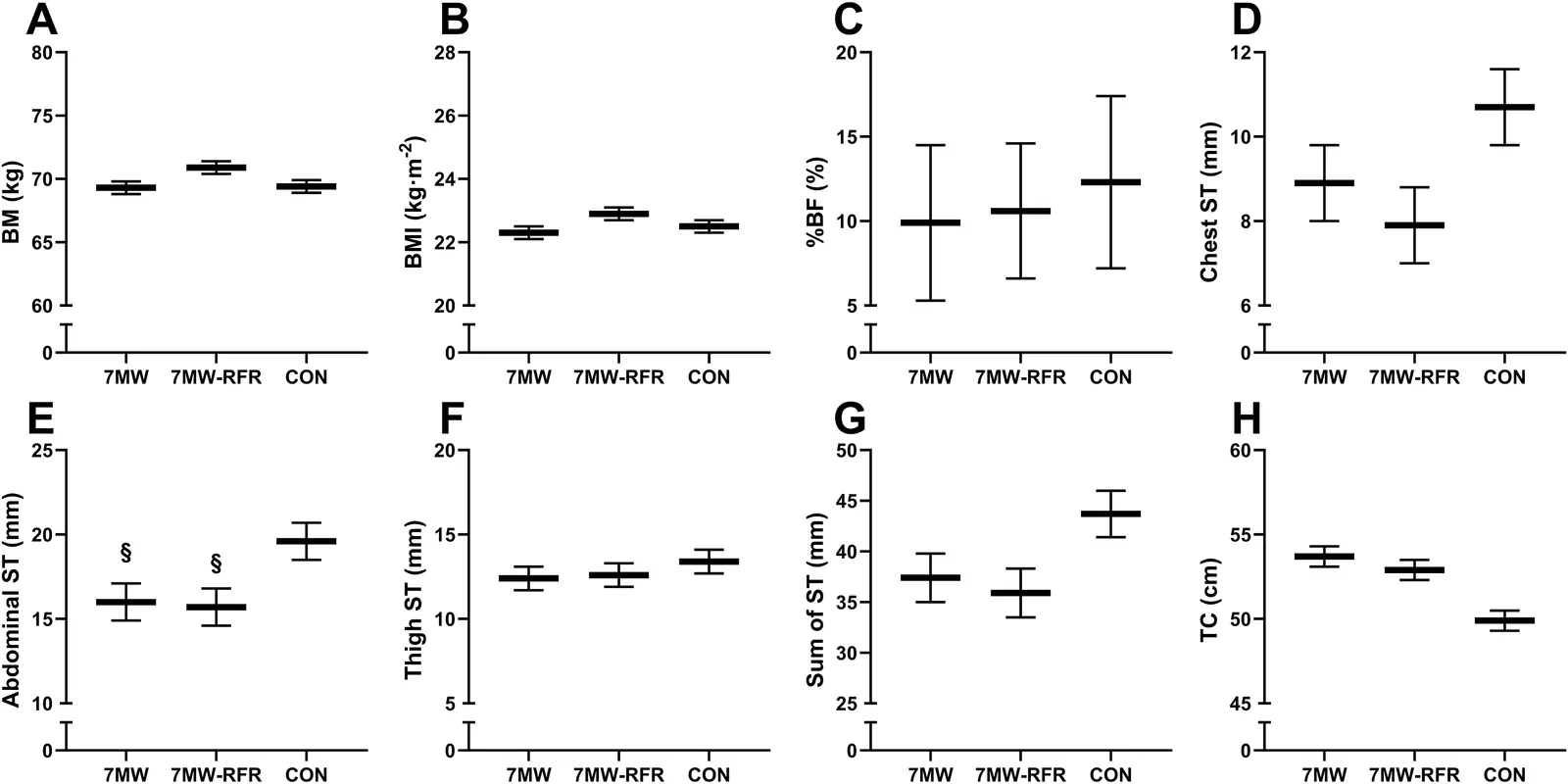
Comparison of the adjusted values of body composition after a 6-weeks intervention in the 7-min workout (7 MW), 7 MW with blood flow restriction (7 MW-BFR), and control (CON) groups. Data are presented as adjusted mean ± standard error (SE). BM: body mass; BMI: body mass index; TC: thigh circumferences; ST: skinfold thickness; %BF: body fat percentage. ^§^*P* < 0.05, compared with CON.

### 3.2 Training intervention

Compliance with the training intervention was 100% and 99% in 7 MW and 7 MW-BFR, respectively. No significant group–time interaction for average s-RPE was found (*F* = 1.328, *p* = 0.263). Both groups showed the average s-RPE of week 4–6 was significantly higher than that of weeks 1–3 (*F* = 31.946, *p* < 0.001; Table 4).

**Table 4:** The comparison of average s-RPE between week 1–3 and week 4–6 in the 7-min workout (7 MW) and the 7 MW with blood flow restriction (7 MW-BFR) groups.

	7 MW (*n* = 11)	7 MW-BFR (*n* = 11)
Week 1–3 (scores)	6.8 (1.6)	7.9 (1.4)
Week 4–6 (scores)	8.3 (1.0)^a^	8.9 (1.0)^a^

Data are presented as mean (standard deviation [SD]).

a *p* < 0.05, compared with week 1–3. s-RPE: session-rate of perceived exertion.

The numbers of repetitions completed during the intervention in the 7 MW and 7 MW-BFR groups are presented in Table 5. Significant group–set interactions for jumping jacks (*F* = 4.065, *p* = 0.025), push-ups (*F* = 7.677, *p* = 0.002), step-ups (*F* = 6.119, *p* = 0.005), squat (*F* = 12.158, *p* < 0.001), triceps dips (*F* = 8.010, *p* < 0.001), and push-up and rotation were found (*F* = 5.578, *p* = 0.017). During week 4–6, the number of push-ups and rotation completed in 7 MW-BFR during the first set was significantly fewer than that in 7 MW (*p* = 0.010, *d* = 1.30, 95% CI: 1–4 times), while the number of push-ups (*p* = 0.024, *d* = 0.99, 95% CI: 0–6 times), step-ups (*p* = 0.039, *d* = 0.99, 95% CI: 1–17 times), squats (*p* = 0.007, *d* = 0.98, 95% CI: 2–8 times), triceps dips (*p* < 0.001, *d* = 1.89, 95% CI: 6–17 times), and push-ups and rotation (*p* = 0.003, *d* = 1.50, 95% CI: 1–5 times) completed in 7 MW-BFR during the second set were significantly fewer than those in 7 MW.

**Table 5:** Comparison of the average number of repetitions (times) in the first set during week 1–3 and in the first and second sets during week 4–6 for each dynamic exercise between the 7-min workout (7 MW) and the 7 MW with blood flow restriction (7 MW-BFR) groups.

	The first set of week 1–3		The first set of week 4–6		The second set of week 4–6	
	7 MW	7 MW-BFR	7 MW	7 MW-BFR	7 MW	7 MW-BFR
Jumping jacks	44 (4)	46 (8)	51 (7)	51 (9)	50 (6)	47 (6)
Push-ups	33 (8)	32 (8)	38 (10)	36 (11)	36 (10)	28 (11)^a^
Crunch	29 (7)	31 (7)	35 (8)	34 (11)	35 (7)	31 (9)
Step-ups	18 (3)	20 (3)	24 (5)	24 (5)	23 (5)	20 (2)^a^
Squats	27 (5)	29 (5)	31 (6)	30 (6)	31 (6)	27 (6)^a^
Triceps dips	29 (7)	28 (8)	34 (7)	30 (10)	32 (6)	23 (10)^a^
High knees running	110 (14)	111 (12)	126 (36)	117 (17)	112 (13)	104 (16)
Lunges	21 (3)	20 (3)	23 (4)	20 (4)	24 (7)	21 (10)
Push-ups and rotation	12 (3)	11 (2)	13 (3)	11 (2)^a^	12 (3)	9 (2)^a^

Data are presented as mean (standard deviation [SD]).

a *p* < 0.05, compared with 7 MW.

## 4 Discussion

The present study demonstrates that a 6-weeks 7 MW intervention can improve aerobic capacity and muscular strength. Furthermore, the incorporation of BFR may yield additional enhancements in lower-limb strength. Nonetheless, no statistically significant changes were observed in lower-limb power following the 7 MW intervention, irrespective of whether BFR was applied. To our knowledge, this is the first study to examine the effects of 7 MW with and without BFR on VO_2_max and muscular fitness in active adults, supporting 7 MW as an easy-implemented and cost-efficient strategy to enhance physical fitness.

Consistent with our hypothesized, the 7 MW program improved VO_2_max in active males when comparing to the CON group, suggesting its utility as an easy-implemented and cost-efficient strategy for enhancing cardiovascular fitness. Although a previous study indicated the intensity of 7 MW only reached the lower boundary of vigorous-intensity in active adults, potentially compromising the adaptation,^11^ we found that a 6 weeks of 7 MW resulted in an increase in VO_2_max of +6.4 ± 5.1%, which is consistent with previous studies.^5^^,^^10^ Previous studies have found increases in VO_2_max during treadmill-based GXT by approximately 11%^10^ and 16%,^5^ respectively, which surpass the improvements observed in the present study. The inconsistency in the degree of improvement may be attributed to differences in the GXT protocols employed. In the present study, a cycling-based GXT was utilized, and the resulting data may be affected by local muscle fatigue in the lower extremities. Nevertheless, it is also important to note that these studies^5^^,^^10^ lacked experimental designs incorporating a control group, potentially compromising internal validity. Additionally, Kuo et al.^6^ demonstrated that the 4-weeks 7 MW program did not lead to improvements in VO_2_max among healthy male participants when compared to the control group, whose baseline VO_2_max values were 8% higher than those observed in the present study. Schmidt et al.^9^ reported that an 8-weeks 7 MW program improved VO_2_max in college-aged females with a baseline VO_2_max of 40.3 mL kg^−1^·min^−1^, but not in males with a VO_2_max of 51.4 mL kg^−1^·min^−1^. Therefore, future studies are needed to clarify the exact effects of 7 MW on aerobic capacity and to determine the optimal training dose for populations with different fitness levels.

Our findings indicated that combining BFR with 7 MW did not yield further improvements in VO_2_max, partially contradicting our hypothesis. A systematic review^24^ reported that incorporating BFR into maximal effort-based HIIT (i.e., SIT or RST) could further enhance aerobic adaptations due to the increased physiological stress. Two potential reasons may contribute to this discrepancy. First, when implementing BFR during maximal effort-based exercise, the impaired performance caused by accelerated peripheral fatigue may reduce training volume.^40^ The present study demonstrated that the total number of completed repetitions was significantly reduced in the 7 MW-BFR group, notwithstanding the comparable s-RPE observed between the two training groups. Second, the relatively low cuff pressure (150 mmHg; ∼30% AOP) might limit the additional benefits of BFR on aerobic adaptation, consistent with Keramidas et al.^41^ who reported no further VO_2_max enhancement after 6-week HIIT combined with 90 mmHg BFR. Patterson et al.^19^ recommended that when using BFR in combination with resistance or aerobic exercise, the cuff pressure should be set to 40%–80% AOP. However, applying excessively high occlusion pressures might cause participants to fail to complete the training. Taylor et al.^27^ indicated that the participants were unable to exert maximal effort during a 30-s sprint with 100 mmHg BFR. To avoid substantial reductions in repetitions and training volume during the 7 MW, higher cuff pressures may be infeasible. Therefore, these findings suggest a potential trade-off between occlusion pressure and the decline in performance during maximal effort-based BFR training. Nevertheless, the relatively low cuff pressure in our study could be considered non-detrimental when incorporated into 7 MW to improve aerobic capacity, as it allows for a reduced training volume while maintaining a comparable level of perceived exertion to that of 7 MW alone.

In the present study, both 7 MW and 7 MW-BFR improved maximal lower-limb strength compared to CON group. These findings may be attributed to the fact that participants were required to perform as many repetitions as possible while engaging in the high-effort, non-restricted bodyweight exercises, potentially resulting in substantial fatigue and increased motor unit recruitment. According to the ACSM position stands,^13^ a near-maximal load (>80% 1RM) is required for trained populations to improve maximal muscular strength. However, our findings indicated that 7 MW may provide a more feasible approach for improving muscular strength in individuals not suited to heavy resistance training or high-impact exercise (e.g., burpees). Additionally, it is worth noting that although no significant difference was observed between the two groups, the 7 MW-BFR intervention demonstrated a large effect size in enhancing maximal muscular strength, whereas the 7 MW exhibited a moderate effect size. Consistent with our findings, Elgammal et al.^28^ indicated that a 4-weeks RST program combined with BFR could facilitate an additional 6.4% gain in lower limb strength. Amani-Shalamzari et al.^42^ also reported that a 3-weeks game-based HIIT with BFR could elicit further improvements in peak torque for knee extension and flexion, compared with those without BFR. As mentioned above, the local hypoxic environment induced by vascular occlusion might accelerate the development of local fatigue thereby increasing the recruitment of type II muscle fibers during exercise, thereby enhancing muscular strength.^18^^,^^19^^,^^24^ This mechanism may help explain why applying BFR during the 7 MW might further improve muscular strength in active adults. Nevertheless, this study is unable to conclusively identify the specific mechanisms responsible for the observed variations in muscular strength outcomes. Therefore, future studies employing larger sample sizes and longer intervention periods are warranted to further examine the effects of combining HICT with BFR on muscular strength and the underlying mechanisms involved.

No significant alterations in body composition were detected after either training protocol, except for the abdominal skinfold thickness in the CON group. Schmidt et al.^9^ also reported no change in body composition after an 8-weeks 7 MW program in males with low baseline %BF (12.4%). In contrast, Matter et al.^8^ observed reductions in %BF and increases in lean body mass after 6 weeks, possibly due to higher baseline %BF in their cohort. These discrepancies may be attributed to variations in participant characteristics. The participants in Matter et al.^8^ had a baseline %BF of 24.1%, which was substantially higher than that in Schmidt et al.^9^ and ours (10.9%BF). Nevertheless, future studies may need to recruit populations with different %BF to clarify the effects of the 7 MW on body composition.

There are several limitations that should be acknowledged. First, an absolute pressure of 150 mm Hg, corresponding to ∼30% AOP, was slightly lower than previous recommendation of 40–80%AOP.^19^ As mentioned above, to avoid substantial reductions in training volume during the 7 MW, the maximum tolerable pressure, i.e., fixed 150 mmHg, was administrated for all participants in this present study. However, this study did not utilize individualized cuff pressures calibrated as a percentage of AOP, potentially leading to heterogeneous degrees of venous occlusion across participants. Consequently, the participants may have been exposed to varying levels of BFR stimulation. Nevertheless, we provide a novel finding that applying lower-pressure BFR during 7 MW can be a potential way to further improve muscular strength. Future studies are warranted to investigate the training effects of prescribing BFR pressure relative to a percentage of AOP. Second, this study was primarily designed to detect changes in aerobic capacity. Therefore, the absence of significant differences observed in power-related or muscular performance measures between 7 MW and 7 MW-BFR should be interpreted with caution. Future study involving larger sample sizes is warranted to clarify the effects of the 7 MW with and without BFR on muscular fitness.

## 5 Conclusions

The 6-weeks 7 MW-based program can improve aerobic capacity and maximal voluntary isometric contraction performance in active adults, with potential additional enhancements in muscular strength when combined with BFR. We suggest that individuals limited by equipment can adopt this cost-efficient and easily accessible approach to improve their physical fitness. Nevertheless, future studies with larger sample sizes are warranted to explore the effects of the 7 MW with and without BFR under different training prescriptions and BFR application strategies (e.g., individualized cuff pressures standardized as a percentage of AOP or longer training period).

## Ethics approval statement

The study was approved by the Research Ethics Committee of National Taiwan Normal University, Taipei, Taiwan (REC no. 202112HM002).

## Data availability statement

The data that support the findings of this study are available from the corresponding author upon reasonable request.

## Authors’ contribution

**CFC and YRY**: Conception and design of study, Analysis and/or interpretation of data, Drafting the manuscript, Revising the manuscript critically for important intellectual content. **CMG and CYL**: Acquisition of data, Analysis and/or interpretation of data, Drafting the manuscript. **CC and CHP**: Acquisition of data, Analysis and/or interpretation of data. The authors declare that no conflicts of interest exist in this article, and that the article was approved for publication by all named authors, and that the work described in the article is original research. The experiments reported in this study were undertaken in compliance with the current laws of the country in which the experiments were performed.

## Funding statement

This study was supported by a grant from National Science and Technology Council, Taiwan (MOST 111-2410-H-003-139-MY2).

## Declaration of competing interests

No conflicts of interest are declared by the authors.
